# Toward a general psychological model of tension and suspense

**DOI:** 10.3389/fpsyg.2015.00079

**Published:** 2015-02-11

**Authors:** Moritz Lehne, Stefan Koelsch

**Affiliations:** Languages of Emotion Research Center, Freie Universität BerlinBerlin, Germany

**Keywords:** tension, suspense, emotion, prediction, music, film, literature

## Abstract

Tension and suspense are powerful emotional experiences that occur in a wide variety of contexts (e.g., in music, film, literature, and everyday life). The omnipresence of tension and suspense suggests that they build on very basic cognitive and affective mechanisms. However, the psychological underpinnings of tension experiences remain largely unexplained, and tension and suspense are rarely discussed from a general, domain-independent perspective. In this paper, we argue that tension experiences in different contexts (e.g., musical tension or suspense in a movie) build on the same underlying psychological processes. We discuss key components of tension experiences and propose a domain-independent model of tension and suspense. According to this model, tension experiences originate from states of conflict, instability, dissonance, or uncertainty that trigger predictive processes directed at future events of emotional significance. We also discuss possible neural mechanisms underlying tension and suspense. The model provides a theoretical framework that can inform future empirical research on tension phenomena.

## INTRODUCTION

Experiences of tension are ubiquitous affective phenomena that pervade many aspects of our lives^1^. Ranging from everyday life events to many leisure activities, tension (and its close relative suspense) is experienced in a wide variety of contexts and constitutes an important ingredient of human emotion. In some contexts, experiences of tension are associated with negative emotions such as fear, concern, or distress, which are generally tried to be avoided; in other contexts, tension is experienced as positive, and can, in fact, be a major motivator to engage in certain activities. The appeal of many forms of media entertainment such as music, film, or literature, for example, often seems to directly derive from their power to evoke feelings of tension and suspense. Similarly, tension is experienced in a multitude of everyday life situations, most typically during the anticipation of uncertain but (potentially) significant events (e.g., medical diagnoses, exams, job interviews, etc.). The omnipresence and potential emotional impact of tension phenomena indicates that they tap into fundamental aspects of human cognition and emotion, and the idea that tension plays an important role in emotion dates back to at least as far as Wilhelm Wundt, who proposed the opposite poles of tension (*Spannung*) and resolution (*Lösung*) as one dimension of affective experience in his three-dimensional model of emotion (Wundt, 1896, 1911). However, recent emotion research has largely neglected the role of tension in emotion, and surprisingly little is known about the psychological mechanisms underlying experiences of tension. Our aim in this article is to re-awaken the interest in tension phenomena and highlight their relevance for psychological research of emotion. We discuss basic psychological principles as well as possible neural mechanisms underlying tension experiences, and propose a general, domain-independent psychological model of tension and suspense. We argue that studying tension phenomena can advance our understanding of emotion by providing new insights into general psychological principles underlying human affect. In particular, studying tension phenomena can shed light on the dynamic, time-varying aspects of emotions, elucidate the relation between emotions and predictive processes, and illuminate the mystery of aesthetic emotions.

Previous research on tension and suspense has mainly focused on specific domains, in particular music (Madsen and Fredrickson, 1993; Bigand et al., 1996; Krumhansl, 1996; Pressnitzer et al., 2000; Lerdahl and Krumhansl, 2007; Farbood, 2012; Lehne et al., 2013a), literature (Rabkin, 1973; Brewer and Lichtenstein, 1982; Gerrig and Bernardo, 1994; Anz, 1998; Fill, 2003; Jacobs, 2011), film (Löker, 1976; Zillmann, 1980; Comisky and Bryant, 1982; Carroll, 1996a; Mikos, 1996; Greifenstein and Lehmann, 2013), and sports (Peterson and Raney, 2008; Knobloch-Westerwick et al., 2009). Though providing valuable insights, domain-specific accounts of tension and suspense run the risk of overestimating the importance of aspects that are idiosyncratic to the particular area of interest, thus losing general mechanisms out of sight^2^. To overcome this limitation, the present paper focuses on domain-independent aspects of tension experiences, arguing that the seemingly different tension and suspense experiences encountered in contexts such as music, film, literature, or everyday life build on the same underlying psychological processes.

Before examining tension phenomena in more detail, a few words about the relation between tension and arousal seem appropriate. Although tension and arousal appear to be closely related, we consider tension phenomena to be a sub-class of states associated with arousal: Whereas a psychological state of high tension is always accompanied by high arousal, a state of high arousal is not necessarily one of high tension or suspense (for example, surprise is associated with a high level of arousal but not necessarily a high level of tension; see also the comparison of tension and surprise in the Section “Expectation, Prediction, Anticipation”). Furthermore, unlike arousal, which is primarily a physiological state that is difficult to study from a purely psychological perspective, tension, and suspense lend themselves better to a psychological investigation like the one described here.

## TENSION AND SUSPENSE AND THEIR RELEVANCE FOR EMOTION RESEARCH

What exactly do we mean by tension? And what can psychological research of emotion gain from studying tension phenomena? First, we define tension and suspense^3^ as affective states that (a) are associated with conflict, dissonance, instability, or uncertainty, (b) create a yearning for resolution, (c) concern events of potential emotional significance, and (d) build on future-directed processes of expectation, anticipation, and prediction (the Section “Key Components of Tension and Suspense Experiences” discusses the different aspects of tension experiences in detail).

Studying tension phenomena can advance psychological emotion research by providing new insights into the following areas in particular:

(1) *Time-varying aspects of emotion.* Emotions are rarely static states and their dynamic, time-varying nature is one of their defining features (Scherer, 2005). However, most experimental emotion research focuses on static aspects during brief emotional episodes (using experimental stimuli that remain relatively constant over time, e.g., affective pictures, facial expressions, words, etc.). Because tension phenomena usually require time to unfold and thus predominantly reflect time-dependent aspects of affective experience, studying these phenomena can help to shift the focus of psychological emotion research to the more dynamic aspects of emotion that are underrepresented in current research.

(2) *The relation of emotion and processes of prediction.* Tension and suspense are closely related to processes of prediction (see the Section “Expectation, Prediction, Anticipation”). These predictive processes have been proposed as a basic principle of human cognition (Gregory, 1980; Dennett, 1996) and brain functioning (Bar, 2007; Bubic et al., 2010; Friston, 2010; Arnal and Giraud, 2012; Clark, 2013), and they may also play a central role in emotion. Being related to both predictive processes and emotion, tension phenomena could provide the “missing link” that may help to bridge the gap between the “cold” cognitive processes of prediction on the one hand and the “hot” processes of emotion on the other hand, thus integrating predictive processes into a general theory of human affect. In this context, studying the brain mechanisms underlying tension experiences from the perspective of the theory of predictive coding (Friston, 2010) seems particularly promising (see Neural Correlates of Tension and Suspense).

(3) *Aesthetic emotions.* Ever since Fechner (1876), aesthetic emotions have attracted the curiosity of psychologists, and recent years have seen a growing interest in the psychology and neuroscience of aesthetic perception and emotional experience (Zeki, 1999; Leder et al., 2004; Silvia, 2005; Jacobsen, 2006; Di Dio and Gallese, 2009; Fitch et al., 2009; Brattico et al., 2013; Juslin, 2013; Nadal and Skov, 2013). However, aesthetic emotions (e.g., emotions evoked in artistic contexts such as music, painting, literature, etc.) remain rather mysterious. Assuming that emotions are generally elicited by events that have some intrinsic significance to the concerns of the individual or, in biological terms, some kind of survival value, emotional responses to abstract, or fictitious events or features of artworks seem paradoxical (but see Darwin, 1871; Dutton, 2009; Menninghaus, 2011, for biological accounts of aesthetic perception and emotion). Apart from other factors contributing to aesthetic experiences (e.g., stimulus complexity, symmetry, or familiarity), patterns of tension and resolution appear to be a key mediator of emotional responses to art forms such as music, literature, or film (cf. Lehne and Koelsch, in press). A better understanding of the mechanisms underlying tension experiences can therefore shed light on the mystery of aesthetic emotions evoked by works of art.

## MEASURING TENSION

**FIGURE 1:**
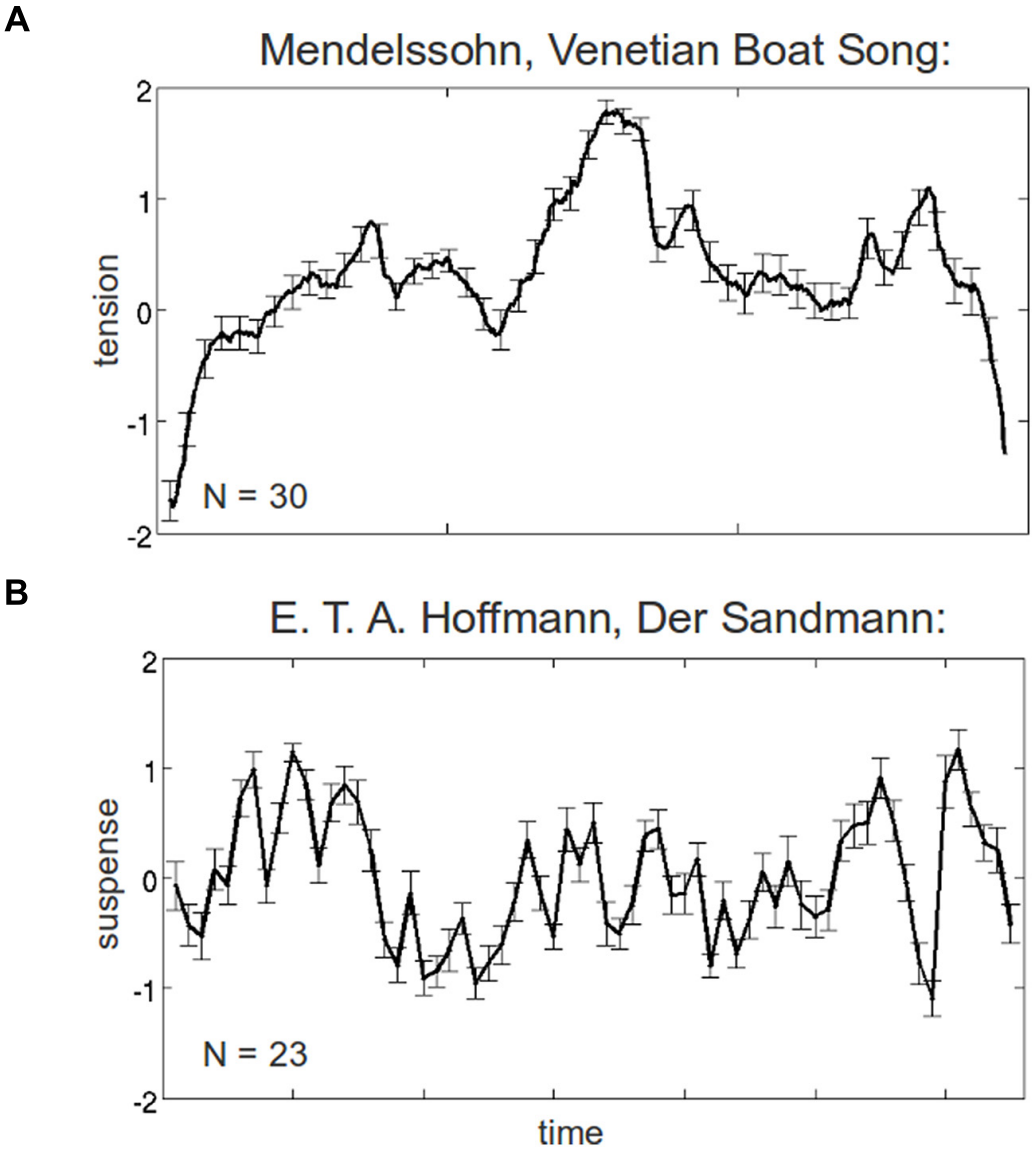
**Average ratings of tension and suspense (standard scores with SE) experienced over the course of **(A)** a music piece (Mendelssohn’s Venetian Boat Song) and **(B)** a literary text (E. T. A. Hoffmann, The Sandman).** For the music piece, tension ratings were acquired using a virtual slider shown on a computer screen that participants continuously adjusted depending on their subjective experience of tension; for the literary text, suspense ratings were acquired at 65 time points during reading of the text.

Experiences of tension are accessible to empirical research by means of subjective rating experiments in which participants give some form of rating reflecting the degree of tension or suspense experienced during exposure to an experimental stimulus (e.g., a piece of music or a literary text). Tension ratings can be acquired continuously over the whole course of the stimulus or they can be collected in a discrete manner at specific time points of the stimulus (see **Figure 1** for two examples). To acquire continuous tension ratings, various kinds of interfaces have been used including spring-loaded tongs (Nielsen, 1983), dial interfaces (Madsen and Fredrickson, 1993), or real or virtual sliders (Krumhansl, 1996; Vines et al., 2006; Farbood, 2012; Lehne et al., 2013a). However, experiments probing tension experiences in music (Madsen and Fredrickson, 1993; Lehne, unpublished data) indicate that different interfaces yield similar results. Moreover, these tension ratings appear to be relatively consistent across and within participants (Krumhansl, 1996; Fredrickson, 1997, 2000).

For discrete measures of tension experiences, the stimulus is usually interrupted several times (unless it is very short) and participants are asked to indicate the amount of tension experienced during the preceding stimulus segment on a rating scale (e.g., in Bigand and Parncutt, 1999; Lerdahl and Krumhansl, 2007). One music study comparing continuous and discrete tension ratings found that both tasks yielded similar results (Lerdahl and Krumhansl, 2007). However, more research is needed to investigate how stimulus interruptions affect tension ratings, or how comparable retrospective ratings are to ratings acquired online during stimulus presentation. Although continuous tension measures may seem preferable to discrete ones, their acquisition is not always feasible because for some stimuli (e.g., written texts) the dual task of focusing attention on the stimulus while simultaneously giving continuous tension ratings can lead to unacceptable quality decreases of the rating data. Moreover, there usually is a temporal lag between the values of continuous tension ratings and the stimulus events they refer to, making it difficult to unequivocally relate stimulus events to exact rating values. The choice between discrete or continuous tension measures, therefore, ultimately depends on the stimuli being used in the experiment and the specific research question.

Furthermore, music research has shown that there can be differences between perceived and felt emotion (Gabrielsson, 2002; see also Konečni, 2008). Because of this, it is important that participants be instructed explicitly whether they are supposed to rate the tension or suspense they *perceive* (i.e., the tension they assume the stimulus is supposed to express) or the tension they subjectively *feel* (i.e., their own actual emotional experience). For example, someone might cognitively acknowledge that a specific movie scene is supposed to induce suspense, without subjectively feeling any suspense.

Experiences of tension and suspense appear to be associated with different physiological responses. Krumhansl (1997) found that the experience of musical tension correlates with finger pulse amplitude, respiration rate, blood pressure, skin conductance, and temperature. Suspense experienced during film scenes appears to be related to increased electrodermal activity and decreased heart rate (Kreibig, 2010). However, there are also reports of increased heart rate during the audiovisual presentation of a suspenseful story (Zillmann et al., 1975). These seemingly discrepant findings with regard to the heart rate may possibly reflect two different physiological processes: a transient vagal suppression of the heart rate as part of an orienting response which is then followed by a longer period of increased heart rate associated with sympathetic arousal.

## KEY COMPONENTS OF TENSION AND SUSPENSE EXPERIENCES

Previously, we defined tension as an affective state associated with conflict, dissonance, instability, or uncertainty concerning events of potential emotional significance that builds on processes of prediction and creates an urge for resolution. The following section examines the different components underlying tension and suspense experiences in more detail (some ideas presented in this section have previously been discussed in Lehne and Koelsch, in press).

### CONFLICT, DISSONANCE, AND INSTABILITY

Tension experiences usually originate from events associated with conflict, dissonance, or instability which create a yearning for more stable, or consonant states. This is, for example, illustrated by the way tension and suspense are created in narrative plots (e.g., in films, novels, theater plays, etc.) or in music. Reaching back to Aristotle’s *Poetics*, in which complication is identified as an integral part of tragedy, a basic “suspense formula” employed by playwrights, fiction writers, and Hollywood directors until the present day is to put the protagonist of a story into a situation of conflict that has to be mastered. This creates tension and suspense experiences in the audience that persist until the conflict is resolved and replaced by a more stable state.

Although seemingly very different from suspense in narrative plots, tension experiences evoked by music (for overviews of musical tension, see Meyer, 1956; Huron, 2006; Farbood, 2012; Koelsch, 2012) appear to be governed by similar principles: musical events associated with dissonance or instability create experiences of tension whereas consonant or stable events are associated with resolution and relaxation. For example, musical chords that deviate from an established tonal key of a musical sequence are perceived as less stable, and accordingly are associated with higher degrees of experienced tension than in-key chords (Lerdahl and Krumhansl, 2007).

Similar to the biological homeostasis of an organism (i.e., its tendency to maintain a stable physiological state in a changing environment), tension experiences thus appear to build on a kind of “psychological homeostasis”—an urge to resolve psychological conflicts and dissonances and strive toward stable states of equilibrium. This idea is related to Festinger’s theory of cognitive dissonance according to which humans try to resolve psychological dissonances to achieve “consonant” cognitive states (Festinger, 1957; Cooper, 2007).

However, in the context of tension and suspense, the general preference for stable states does not mean that tension experiences are always associated with negative emotion (such as anxiety), nor that stable states of equilibrium are always favored over states of tension. To the contrary, events associated with tension may be perceived as positive because they promise excitement and intense emotional experiences that are usually not experienced in states of constant equilibrium. This is especially true in contexts such as music, film, or literature in which seemingly negative tension experiences can be experienced as positive and rewarding because they usually lack any negative real-life implications (cf. Levinson, 1997). Moreover, the state achieved after having gone through an experience of tension may be preferable to the one before the tension experience, thus sometimes justifying the deliberate exposure to negative tension experiences. For example, tension is often associated with situations in which an individual’s model of the world (i.e., previous knowledge, and expectations) is challenged, providing an environment for learning in which the model of the world is expanded.

### UNCERTAINTY

Another important building block of tension experiences is uncertainty (although its exact role in mediating tension and suspense is a matter of debate, see the Section “The Paradox of Suspense”). Obvious instances of uncertainty triggering experiences of tension are real life examples in which anticipated future events with uncertain but potentially highly significant outcomes can create strong tension experiences (e.g., a medical diagnosis, an important job interview, a rendezvous, etc.). Similarly, in narrative plots such as novels or movies, uncertainty about the unfolding of events of the plot creates suspense. The uncertainty associated with tension experiences—whether in real life or in narrative plots—often takes the form of an implicit or explicit question (e.g., the classic “Whodunit?” in a detective story), triggering an experience of tension that resolves when an answer to the question is provided. Uncertainty can take on different forms, for example, there can be uncertainty about *what* will happen, *how* it will happen, *when* it will happen, or *if* it will happen.

### EXPECTATION, PREDICTION, ANTICIPATION

A key component underlying tension and suspense experiences are future-directed processes of expectation, prediction, and anticipation^4^. As events unfold in time (e.g., in real life, fictional worlds, or music), they are constantly evaluated against a background of predictions that is continuously updated during the temporal evolution of events. Anticipated events of emotional significance (see Emotional Significance of Anticipated Events) can then generate experiences of tension or suspense. The resulting tension experiences are closely related to the emotions of hope and fear (cf. the concept of Prospect emotions described in Ortony et al., 1988): anticipated events with positive valence elicit emotions of hope, whereas negative events create fear (or anxiety if the anticipated events are more diffuse and not clearly specified). In typical tension experiences, both emotions can co-occur because often both positive and negative outcomes are possible. In fact, the degree of experienced tension appears to be directly related to the range of anticipated events and their emotional valence. That is, the experience of tension is more intense if the range of anticipated outcomes varies from highly positive to highly negative events, as opposed to, for example, slightly positive to neutral events (see Toward a Psychological Model of Tension and Suspense).

The formation of predictions requires explicit or implicit knowledge such as learned concepts, categories, schemata, or scripts (Bartlett, 1932; Rosch et al., 1976; Schank and Abelson, 1977; Rumelhart, 1980). Furthermore, both long-term and short-term knowledge are relevant for the formation of predictions. Predictive processes in music, for example, build on long-term knowledge that is acquired over years of exposure to a musical system (e.g., Western tonal music), as well as short-term knowledge that is acquired during the exposure to a specific music piece (Pearce and Wiggins, 2012; Rohrmeier and Koelsch, 2012; Tillmann et al., 2014). Musical events generating strong expectations for upcoming events are then potential triggers of tension. Notably, these expectations need not necessarily be fulfilled but can be violated, thus generating surprise. Tension and surprise thus can be complementary if a sequence ends with a surprising event (e.g., a joke). This is in accordance with Huron who states that “tension is almost the exact opposite of surprise” because “surprise happens after events; tension happens before events” (Huron, 2006, p. 307). However, if a surprising event does not terminate the sequence (e.g., a deceptive cadence in a musical piece), but rather delays the final event of the sequence, the surprising event might even further increase tension (however, this assumption remains to be clarified by empirical research). The build-up, fulfillment, and violation of listeners’ expectations has been identified as an important mechanism for the evocation of emotions in music (Juslin and Västfjäll, 2008; Vuust and Frith, 2008; Koelsch, 2012).

During the formation of predictions, the probability of anticipated events also plays an important role and shapes the resulting experience of tension or suspense. However, the exact relation between anticipated probabilities of future events and experienced tension remains largely unclear (but for the special case of narrative plots and film suspense, see Comisky and Bryant, 1982; Gerrig and Bernardo, 1994; Carroll, 1996a).

Importantly, by building on implicit knowledge, predictions can be established automatically (i.e., in the absence of volition or awareness). In music, for example, statistical regularities can be acquired implicitly through exposure to a musical system (Rohrmeier and Rebuschat, 2012), and musical expectancy violations are processed based on such implicit knowledge even in the absence of attention (Koelsch et al., 2002).

### THE PARADOX OF SUSPENSE

If tension experiences build on an urge for uncertainty reduction and processes of expectation, prediction, and anticipation, this raises the question as to how tension can be experienced over repeated exposures to a stimulus. Assuming that uncertainty is resolved after the first exposure, subsequent exposures should have lost their power to create tension or suspense. However, this does not always appear to be the case, and in some contexts, tension experiences seem to be resistant against the loss of uncertainty associated with repeated exposures. A piece of music can be listened to hundreds of times while still conveying feelings of tension and resolution, and—at least according to some authors (see Gerrig, 1989; Carroll, 1996b)—the narrative plots of novels or movies can be experienced as suspenseful even in the absence of uncertainty after several readings or viewings. Moreover, music pieces, novels, or movies often conform to genre-specific standards that make it possible to predict outcomes with high accuracy (in a typical Hollywood movie, the “good guys” usually win, a Beethoven sonata will end on a consonant stable chord, etc.), thus putting the role of uncertainty as a mediator of tension into question. This apparent contradiction known as the *paradox of suspense* has spurred much discussion, and conflicting accounts have been put forward as possible solutions^5^. While some authors question the possibility of experiencing suspense in the absence of uncertainty (Yanal, 1996), others have argued that the experience of suspense after repeated exposure to a narrative plot derives from the sympathy with the characters (who themselves are uncertain about future events, Skulsky, 1980), or that immersing into a suspenseful story for repeated times involves a game of make-believe, in which a kind of feigned uncertainty, not actual uncertainty, causes the experience of suspense (Walton, 1978). A related idea was proposed by Gerrig (1989) who postulated that external events automatically are processed with the “expectation of uniqueness,” i.e., they are perceived as if they have never been encountered before.

Furthermore, although there may be certainty about what will happen after repeated exposure to, for example, a suspenseful movie, viewers may not recall exactly when certain events happen, and these temporal uncertainties may be the main factor triggering suspense experiences after repeated exposure to a stimulus. Distinguishing between felt and perceived tension or suspense (see Measuring Tension) may also account for the resilience of some suspense experiences because repeated exposures may decrease the level of felt suspense but not that of perceived suspense.

With regard to music, empirical evidence suggests that unexpected musical events are processed pre-attentively and automatically with regard to their music-syntactic function (Koelsch et al., 2002). Due to this automatic processing, a musical event can be experienced as music-syntactically unexpected, despite familiarity with the piece (which should render all musical events expected). This would account for the experience of musical tension in the absence of uncertainty (see also the distinction between schematic and veridical expectations in music, Bharucha, 1994).

Despite the resilience of tension experiences over repeated exposures to some stimuli, we think that in most cases the reduction of uncertainty, *ceteris paribus*, leads to a considerable decrease of tension or suspense. Watching a recording of the soccer World Cup finals is clearly less suspenseful when knowing the end result of the game, and a suspenseful movie is rarely watched twice in close succession, indicating that parts of the suspense experience do get lost after repeated exposure. Nevertheless, the paradox of suspense indicates that the exact role of uncertainty in creating experiences of tension and suspense is still not fully understood and remains to be investigated more closely.

### EMOTIONAL SIGNIFICANCE OF ANTICIPATED EVENTS

Importantly, anticipated events need to be relevant to the concerns of the individual, i.e., they have to have some emotional significance, in order to generate tension or suspense. Apart from expecting specific events to happen, someone experiencing tension or suspense usually also *wants* a specific event to happen (or not to happen; cf. Anz, 1998). In fact, the amount of tension experienced appears to depend directly on the significance or desirability of anticipated events. Event outcomes promising great reward (e.g., winning the lottery) or portending great pain or suffering (e.g., a medical diagnosis) can elicit strong experiences of tension whereas events that are largely irrelevant to the concerns of the individual usually fail to create tension or suspense. Of course, the emotional significance of events can differ largely between individuals. The idea that tension experiences depend on the significance of anticipated events is in accordance with cognitive theories of emotions, according to which emotions result from events being evaluated in relation to concerns (for an overview of cognitive approaches to emotion, see Oatley and Johnson-Laird, 2014). It also concurs with Reisenzein’s (2009) computational belief-desire theory of emotion, which states that emotions depend on an individual’s beliefs and desires (Reisenzein, 2009).

Whereas the relevance of an event’s emotional significance is relatively obvious for most real life examples of tension (job interviews, exams, etc.), this is not the case for the tension experiences created by many forms of media entertainment: the fictitious events of a movie or a novel, or the musical events of a Beethoven symphony appear to lack any direct relevance to our lives, yet they can trigger powerful experiences of tension. Nevertheless, even for fictitious events of a narrative plot, it is important that they become significant to the reader or viewer, and many writers or movie directors go through great lengths to make audiences care about the events of the plot (e.g., by portraying the protagonist as likable, addressing moral values of the reader, etc.). This can result in processes of identification and empathy with characters of the plot that could account for the emotional responses evoked by narrative plots (cf. Hogan, 2010; Oatley, 2012).

### LACK OF CONTROL

Apart from the factors discussed above, a lack of control, i.e., an inability to influence the course of events, often contributes to experiences of tension. This lack of control is brought about by a temporal distance between the initiating event triggering the tension experience and the event that resolves the tension. During the time interval between these two events, there is usually not much that can be done except for waiting for the tension to resolve (in the best case the time of resolution can be influenced). This means that during the actual tension experience any action tendencies are rendered largely ineffective because the course of events cannot be changed, and this may induce a feeling of helplessness that can add to the experience of tension. This, of course, does not mean that tension experiences are devoid of action tendencies—to the contrary, tension experiences can evoke strong impulses to act and these impulses may prepare the organism for adequate behavioral responses in the moment when tension resolves.

The inability to influence events often becomes apparent in suspenseful movie scenes that build on a disparity between the knowledge of a character of the movie and the knowledge of the viewer (e.g., the viewer may be made aware that the protagonist is in huge danger while the protagonist is completely oblivious of this danger). The resulting lack of control over the situation may contribute to feelings of tension and suspense and can create strong action tendencies (viewers, for example, may have the strong urge to utter warnings to the protagonist, despite knowing that this is pointless).

### TEMPORAL ASPECTS

Tension experiences can be observed at different temporal levels. They can span large time intervals encompassing, for example, the complete plot of a novel but they can also be observed at smaller, microstructural levels. In a written text, for example, a single sentence which is very long, and in which subject and verb are separated by various subordinate clauses or parenthetical statements (e.g., by making ample use of relative clauses, brackets, dashes, etc.), thus taxing the working memory load of the reader—longer sentences usually require more elements to be kept active in memory—and delaying syntactic integration necessary for understanding the sentence, can create tension. The different temporal levels (such as sentences, scenes, and entire plots) can interact, thus potentially amplifying the tension experience.

The previous example also illustrates that the temporal distance between the initiating event creating the tension and the moment in which tension resolves influences the tension experience. It has been proposed that delaying the resolution of the tension intensifies the tension experience (de Wied, 1995), however, there is a scarcity of empirical research investigating the relation between deferment of the resolution and experienced tension. Investigating the temporal aspects of tension experiences more closely would substantially contribute to a better understanding of tension and suspense (see Future Directions).

## TENSION AND EMOTION

We already mentioned the relation between tension experiences and emotions of fear and hope (i.e., fear of an undesirable outcome and hope for a desired outcome). Because tension experiences are directed at future events of emotional significance, tension often precedes other emotions such as joy, pleasure, sadness, or disappointment and can thus be conceived of as a diffuse affective antecedent to more discrete emotional reactions. Generally, the more divergent the emotional valence of anticipated events is, the more diffuse and unspecific the associated tension experience tends to be. For example, in situations, in which both highly positive or negative outcomes are possible (e.g., betting a thousand dollars on the result of a coin flip), tension experiences may have neither a clear positive nor negative valence (although in this specific coin flip example, tension is probably experienced as more negative due to people’s tendency for loss aversion, cf. Kahneman and Tversky, 1984). On the other hand, when the affective valence of possible outcomes is negative and the best outcome is just a preservation of the status quo, associated tension experiences also tend to be negative (causing anxiety or stress), whereas if a positive outcome is anticipated and the worst outcome is a preservation of a neutral status quo the tension experience tends to be positive. The emotional valence of tension experiences is therefore often defined by the emotional valence of the anticipated resolution. Contrary to that, one may argue that tension experiences are largely associated with negative emotion, and that people deliberately expose themselves to tension experiences only for the sake of experiencing the positive resolution of tension. Accounts such as Berlyne’s (1960) arousal-jag theory or Zillmann’s (1980) excitation-transfer paradigm, for example, assume that the intensity of the pleasure experienced during the resolution depends on the intensity of the (negative) tension experienced prior to the resolution. However, even in the absence of a positive resolution, tension experiences can be enjoyed, particularly in forms of media entertainment such as film or literature that often lack a positive resolution (e.g., tragedies, some horror movies, etc.). The emotional intensity or “thrill” of the tension experience may thus be appreciated for its own sake, and the fictitious events of narrative plots provide an opportunity to experience intense emotions associated with situations not commonly encountered in everyday life and without any (potentially negative) real life consequences (cf. the “pleasure of being moved” discussed in Hanich et al., 2014).

In its most diffuse form, tension can just arise from the expectancy that “something” significant will happen. Depending on what then actually happens, tension resolves into positive or negative emotions. If something completely unexpected happens, this can lead to surprise or amusement, e.g., when expecting something significant to happen that then resolves into something trivial (cf. the false-alarm-theory of humor and laughter; Ramachandran, 1998).

It also seems worthwhile to examine the relation of tension experiences and flow (Csíkszentmihályi, 1990)—the state of being completely absorbed in an activity (e.g., reading, watching a movie, listening to or performing music, sports, etc.). Tension and suspense, like flow, are associated with strong immersive experiences in which attention is highly focused (it is, for example, remarkable how long, especially in comparison to other stimuli, a suspenseful novel or movie can capture one’s attention). A better understanding of the psychological mechanisms underlying tension and suspense could make it possible to deliberately create these experiences of minimal distraction and highly focused attention associated with flow.

## TOWARD A PSYCHOLOGICAL MODEL OF TENSION AND SUSPENSE

**FIGURE 2:**
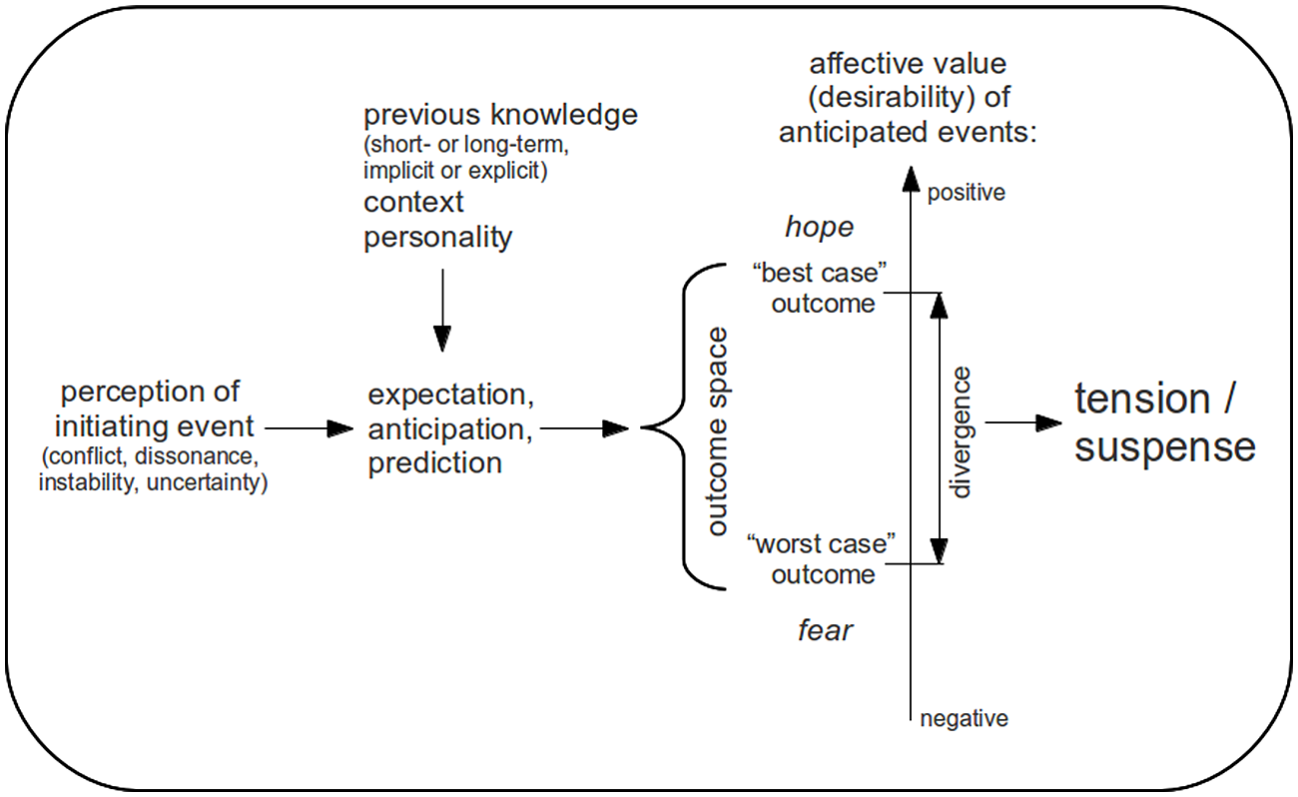
**Tension model.** The perception of an initiating event associated with conflict, dissonance, instability, or uncertainty triggers future-directed processes of expectation, anticipation, and prediction (depending on previous knowledge, context, and personality factors) generating a space of anticipated outcome events that vary with regard to their affective values/desirability. A divergence between the affective values of anticipated outcomes (i.e., a differing desirability of outcome events) leads to the subjective experience of tension (with positive outcomes being associated with hope and negative outcomes with fear).

**Figure 2** shows a model of tension and suspense based on the points discussed in this article. According to this model, experiences of tension originate from the perception of an initiating event that is associated with conflict, instability, dissonance, or uncertainty which triggers future-directed processes of prediction, expectation and anticipation (modulated by previous knowledge, situational factors, or personality, see below). These predictive processes create a space of possible outcome events (note that these anticipated outcome events can be conscious or unconscious, and more or less specific). A divergence between the affective values of anticipated events (i.e., their desirability) then results in an experience of tension. More specifically, we propose that the intensity of tension experiences increases as the variability of the affective values of anticipated outcomes increases. That is, anticipated events whose affective values are highly variable (e.g., ranging from very positive to very negative events) are associated with higher degrees of experienced tension than events whose range of affective values is smaller. It remains to be investigated how exactly the dispersion of the affective values of anticipated events affects the tension experience (e.g., whether tension is affected more by the range of affective values or by their variance). Furthermore, the perceived probabilities of anticipated events influence the tension experience, and a closer investigation of this effect of perceived probabilities on tension experiences might eventually lead to a quantitative mathematical model of tension and suspense.

Note that the most positive or negative event of the outcome space is often just the maintenance of the status quo whereas the others are more positive or negative events (for example, tension can be created by a mismatch between a desired state and the actual state of the world). Outcomes that have a negative affective value are associated with fear whereas positive outcomes can evoke feelings of hope. Initiating and outcome events can coincide, i.e., an outcome event can be the initiating event of a new tension process, thus creating a succession of different tension experiences that give rise to a dynamic flow of tension and resolution (e.g., in a piece of music or in a movie).

Importantly, the anticipated events of the outcome space as well as their affective values and perceived probabilities can largely differ between (and within) individuals because the predictive processes generating the outcome space depend on factors such as previous knowledge, personal values, mood, the context, in which events occur, attention, or personality (e.g., whether someone generally has a more optimistic or pessimistic outlook on the future).

## NEURAL CORRELATES OF TENSION AND SUSPENSE

Investigating tension phenomena from a neuroscientific perspective can provide insights into general mechanisms of human brain functioning, in particular the ones associated with emotion and predictive processing. Vice versa, neuroscientific findings can inform psychological theories of tension.

First empirical research investigating brain processes underlying the experience of tension in music indicates that musical tension is associated with neural activity in the lateral orbitofrontal cortex, and that increases in experienced tension (in comparison with tension decreases) are related to activity in the (superficial) amygdala (Lehne et al., 2013b). This is consistent with studies showing that harmonic expectancy violations in music (which are highly relevant to experiences of tension, see Expectation, Prediction, Anticipation) also activate the lateral orbitofrontal cortex (Koelsch et al., 2005; Tillmann et al., 2006) and the amygdala (Koelsch et al., 2008). In addition, other music studies indicate that expectation processes are related to brain areas in the basal ganglia associated with reward processing, particularly the striatum (Salimpoor et al., 2011; Seger et al., 2013).

Investigating immersion in reading (i.e., the feeling of “getting lost” in a text), which is closely related to suspense, a study by Hsu et al. (2014) indicates that immersion during emotional text passages is associated with activation of the mid-cingulate cortex, possibly reflecting empathic processes with protagonists of the plot. Further evidence from a study investigating suspense during reading suggests that the experience of suspense recruits brain areas related to theory-of-mind processing such as the medial prefrontal cortex (mPFC) and the temporo-parietal junction (TPJ) as well as areas in the premotor cortex (Lehne et al., submitted). Such premotor cortex activations have been implicated with action and event prediction (Schubotz, 2007), underlining the connection between experiences of suspense and predictive processes. With regard to these predictive processes, the connection of psychological tension theories with Bayesian accounts of brain functioning such as predictive coding and the free energy principle (Friston and Kiebel, 2009; Friston, 2010) may prove useful. According to predictive coding theories, perception, action, and learning are essentially based on the minimization of prediction errors, surprise, and uncertainty, i.e., it is assumed that the brain constantly generates predictions at different levels of the processing hierarchy that are compared with input from lower levels of the hierarchy (e.g., sensory input). If there is a mismatch between (top–down) predictions and (bottom–up) input, this results in a prediction error. If such a prediction error occurs, predictions are updated or behavior is changed in such a way that predictions are fulfilled, thus minimizing future prediction errors, surprise, and uncertainty. Although these predictive processes most likely also have an emotional component, predictive coding and emotion are only beginning to be integrated into a common theoretical framework (Joffily and Coricelli, 2013). As discussed above, tension and suspense are closely connected with processes of expectation and anticipation and thus appear to rely on very basic brain mechanisms associated with predictive processing. At the same time, tension phenomena can evoke intense emotional responses. Studying neural correlates of tension and suspense would therefore be useful when developing a theory of human brain function integrating cognitive processes of prediction with affective emotional processes. For the special case of music, an integration of predictive processes, emotion, and underlying brain processes has recently been proposed by Gebauer et al. (2012), who relate music-evoked emotions to so-called “pleasure cycles” which are distinguished by different phases of wanting, liking, and learning (for details, see Georgiadis and Kringelbach, 2012; Kringelbach et al., 2012) and which depend on listeners’ expectations and their fulfillment or violation. The different phases of these pleasure cycles are put into relation with neuronal processes of the dopaminergic reward system representing expectations and prediction errors according to the theory of predictive coding. Art forms such as music, film, or literature thus appear to be capable of evoking activity within these brain circuits associated with prediction and reward. Therefore, studying the underlying brain mechanisms more closely would advance our understanding of the aesthetic emotions evoked by works of art.

Electroencephalography (EEG) studies investigating tension and suspense are scarce. However, event-related potentials such as the P300 and the contingent negative variation (CNV) which have been implicated in processes of anticipation (Verleger et al., 1994; Goldstein et al., 2006; Pfabigan et al., 2014) may play a role in tension and suspense experiences.

Tension experiences also share many of the components that are relevant to the perception of risk (e.g., in decision-making) such as uncertainty, predictive processing, and emotion. Brain regions associated with tension experiences may therefore overlap with regions that have been implicated in risk processing such as the anterior insula or dorsomedial and dorsolateral prefrontal cortex (Mohr et al., 2010). The same is true for brain regions associated with anticipatory processes such as the dorsal striatum (cf. Salimpoor et al., 2011), which have mostly been studied in the context of reward processing (Schultz et al., 1997; O’Doherty, 2004; Wise, 2004; Knutson and Cooper, 2005).

Apart from providing insights into the neural mechanisms underlying tension experiences, neuroscientific methods may also open new perspectives for objectively quantifying the amount of tension or suspense evoked by an experimental stimulus (e.g., a movie excerpt, a piece of music, etc.). Studies by Hasson et al. (2010) have shown that the intersubject synchronization of cortical activity, measuring to which degree brain activity correlates between different individuals, varies between different kinds of audiovisual stimuli, with the highest synchronization observed for suspenseful movies (Hitchcock’s “Bang! You’re dead” or Leone’s “The good, the bad and the ugly”). Similarly, Nummenmaa et al. (2012) have shown that emotional movie scenes are associated with increased intersubject correlations of brain activity. The intersubject synchronization of brain activity could therefore possibly serve as an indicator of how much suspense a stimulus evokes on average over a group of participants.

## FUTURE DIRECTIONS

The model proposed in this article should be regarded as a first step toward a psychological theory of tension and suspense. It provides a starting point from which experimental hypotheses can be generated and tested in empirical studies, which may then motivate further refinements of the model. In particular, future research is needed to specify how the perceived probabilities of anticipated events affect the subjective experience of tension. Furthermore, the role of uncertainty in generating experiences of tension and suspense is unclear and remains to be resolved by investigating how tension experiences change over repeated exposures to an experimental stimulus.

Moreover, the model still lacks a temporal component. Because tension and suspense unfold in time, investigating temporal aspects of tension experiences would provide further insights into the psychological mechanisms underlying experiences of tension. More specifically, it remains to be investigated how the subjective experience of tension develops between the perception of the initiating event and the anticipated outcome event. Do tension and suspense increase linearly between these two events? Does increasing the delay of the outcome event also increase tension or is there a time point after which tension decreases again? Is there an “optimal” temporal distance between the initiating event and the anticipated outcome event that would maximize the tension experience? Empirically exploring these and similar questions would add to a better understanding of tension experiences.

We also mentioned that the factors determining the degree of experienced tension (such as the affective values of anticipated events and their perceived probabilities) are highly subjective and context-dependent. It is therefore worthwhile to explore how personality, mood, or situational factors shape experiences of tension. Related to that, it remains to be investigated more closely in which contexts tension is experienced as positive, and when as negative. Investigating neural mechanisms underlying tension experiences, in particular the ones related to predictive processes, could further add to a psychological theory of tension and suspense.

## CONCLUSION

In the present paper, we explored psychological mechanisms underlying experiences of tension and suspense. We discussed the relevance of tension phenomena for emotion research (e.g., aesthetic emotions), identified key components of tension experiences, proposed a general psychological model of tension and suspense, and reviewed possible neural mechanisms underlying tension experiences. According to the model, tension experiences originate from events associated with conflict, instability, dissonance, or uncertainty which trigger processes of prediction. The divergence between the affective values (i.e., the desirability) of anticipated events then results in experiences of tension. The model provides a starting point from which open questions concerning the psychological underpinnings of tension phenomena can be addressed. In particular, future research could investigate how temporal aspects shape tension experiences (e.g., the delay of resolution moments), how the probability of anticipated events influences tension, and how situational and personality factors contribute to tension experiences. Furthermore, the paradox of experiencing tension and suspense over repeated exposure deserves a closer investigation. Last, integrating affective experiences of tension into neural theories of predictive coding offers promising avenues for future research.

## Conflict of Interest Statement

The authors declare that the research was conducted in the absence of any commercial or financial relationships that could be construed as a potential conflict of interest.

## Footnotes

1 By experiences of tension we refer to psychological experiences of tension (in contrast to purely physiological tension such as muscular tension, although both kinds of tension can interact).

2 For example, in theories of suspense in narrative plots (e.g., movies or novels), concern about the well-being of a sympathetic protagonist is often discussed as a key element of suspense (Skulsky, 1980; Zillmann, 1980). However, this aspect of suspense in narrative plots obviously does not generalize to tension phenomena in music or many tension experiences in everyday life. On the other hand, specific aspects of musical tension are irrelevant to film suspense.

3 Notice that, despite being often used interchangeably, the terms tension and suspense sometimes denote slightly different experiences. Whereas suspense usually involves the anticipation of two clearly opposed outcomes (e.g., the heroine of a movie killing the villain as opposed to the villain killing the heroine), the term “tension” often denotes a more diffuse state of anticipation, in which anticipated events are less specific. However, there is no clear-cut boundary between the two concepts, because anticipated events can be situated on a continuum ranging from very unspecific (expecting “something” to happen) to very specific. Throughout the article, we will try to show that both concepts build on the same underlying psychological principles.

4 Although often used interchangeably, there are subtle conceptual differences between expectation, expectancy, prediction, and anticipation. We use the term “expectation” to refer to distinct instances of future events (i.e., expecting a specific event to happen), whereas the terms “expectancy” and “anticipation” are used to denote predictive processes that are more diffuse and less specific. “Prediction” is used to refer to all processes directed at future events, whether specific or unspecific (for accounts on terminology, see also Bubic et al., 2010; Rohrmeier and Koelsch, 2012).

5 Note that for most real life tension experiences (i.e., the ones not pertaining to narrative plots or music), the paradox of suspense is less relevant, because the impossibility of time travel usually impedes the repeated exposure to real life events.
